# A Virtual Pulmonary Rehabilitation Program to Address Gaps in Pulmonary Rehabilitation: Retrospective Observational Study

**DOI:** 10.2196/85813

**Published:** 2026-11-02

**Authors:** Alexander Beschloss, Andrey Ostrovsky

**Affiliations:** 1 Department of Medicine, Massachusetts General Hospital, 55 Fruit street, Boston, MA, 02114, United States, 1 6177262000; 2 Social Innovation Ventures, Washington, DC, United States

**Keywords:** pulmonary rehabilitation, telemedicine, digital health, virtual care

## Abstract

**Background:**

Traditional brick-and-mortar pulmonary rehabilitation (PR) programs remain substantially underused, with only 2.7% of Medicare beneficiaries completing PR within 12 months following hospitalization for a COPD exacerbation. Given the critical role of PR in improving exercise tolerance, quality of life, and reducing readmissions, innovative delivery models are needed to address this massive utilization gap. Virtual PR platforms have emerged as a potential solution, offering scalable, and home-based rehabilitation.

**Objective:**

This study aimed to evaluate the impact of a novel virtual PR program on functional, psychosocial, and cardiometabolic outcomes among adults with COPD.

**Methods:**

This retrospective observational study analyzed outcomes among patients enrolled in a structured, multidisciplinary virtual PR program between 2022 and 2024. Participants completed individualized exercise and education sessions remotely, supported by a multidisciplinary team including Physicians and Clinical Exercise Physiologists. Baseline and postprogram measures were compared using Cohen *d* to assess effect sizes across clinical domains. Primary outcomes included changes in maximum metabolic equivalents (METs), Duke Activity Status Index (DASI), and Patient Health Questionnaire-9 (PHQ-9) scores. Other measures included changes in blood pressure (systolic and diastolic), smoking status, and prevalence of hypertension.

**Results:**

Among 2444 patients completing the program, the largest effect size improvements were observed in maximum METs achieved, DASI, and PHQ-9 scores. The program was also associated with decreased smoking rates and a lower prevalence of hypertension post intervention. Notably, 91% of the studied population self-identified as having a “fixed income,” underscoring the potential for virtual PR to extend access to patients with lower incomes in the United States.

**Conclusions:**

Virtual PR appears to be a promising and potentially more scalable alternative to traditional facility-based PR for patients with COPD. Significant improvements in exercise capacity, mood, and cardiovascular parameters suggest clinical benefit, while reductions in smoking and hypertension further highlight potential for broader health impact. Given the high proportion of participants on fixed incomes, virtual PR may represent an effective strategy to expand access and equity in chronic respiratory care delivery across the United States.

## Introduction

Pulmonary rehabilitation (PR) is a comprehensive, patient-tailored intervention that includes exercise training, education, and behavioral support to improve the physical and psychological condition of individuals with chronic respiratory disease [1]. PR improves patient symptoms, exercise capacity, emotional health, and mortality; however, many patients in the United States have faced challenges with sufficient access to it [1-4].

Notably, there are substantial geographic and travel time disparities in access to PR [5]. The American Thoracic Society recently published an editorial highlighting some of the challenges including travel time and racial disparities [6,7]. Unfortunately, only 2.7% of Medicare patients hospitalized for chronic obstructive pulmonary disease (COPD) receive PR within 12 months of discharge [8]. Even once a patient is enrolled, it takes a median of 95 days for them to receive their first PR session [8].

Virtual PR, that is, PR delivery through telehealth platforms, may be a solution to these challenges, which may include video-based exercise training, remote monitoring, education, and behavioral support conducted in a patient’s home environment [9]. In fact, a 2017 prospective blinded randomized controlled trial (RCT) found that supervised home-based PR improved endurance exercise capacity and self-efficacy in patients with COPD compared to usual medical management [10]. PR has also been found to be associated with net cost saving and improvements in patient’s Quality Adjusted Life expectancy in a 2022 societal perspective analysis [11].

The systematic review and meta-analysis by Li et al [12] identified that pulmonary telerehabilitation and traditional center-based PR had comparable average effects on exercise capacity, as determined by 6-minute walk distance, suggesting the promise of this tool; however, they did not investigate the additional benefits that be associated with secondary outcome measures (eg symptomatology, mental health, and more).

However, there is currently limited real-world evidence on outcomes data, safety, engagement, demographics and patient-reported measures to support the adoption of these technologies at scale; therefore, we measured and evaluated these aforementioned measures to investigate the safety and efficacy of virtual PR.

## Methods

### Population and Study Design

This is a 2444-patient retrospective cohort study (2022‐2024) that investigates outcome measures across all patients for whom the PR protocol was initiated (noting that the American Association of Cardiovascular and Pulmonary Rehabilitation [AACVPR] considers 10 sessions to be the threshold for PR “completion”). The study population was composed of patients who met at least one American Thoracic Society indication for PR: the top three diagnoses were COPD, (82.4%); dyspnea, unspecified (4.7%); and emphysema (3.7%). Outcome data include the number of days from enrollment to the first session, difference in mobilization assistive devices needed, resting systolic blood pressure (SBP) and diastolic blood pressure (DBP), BMI, the percentage of sessions with shortness of breath (SOB) or chest pain (CP), resting heart rate, medication adherence, Duke Activity Score (DASI), Patient Health Questionnaire-9 (PHQ-9) score, Sit-to-Stand test score, and maximum metabolic equivalents (METs) achieved. Regarding mobilization devices, we normalized them to a numerical score as follows to permit quantitative analysis: 3=wheelchair, 2=walker, 1=cane, and 0=N/A (not applicable). We also performed subgroup analysis to measure changes in PHQ-9 score and BMI in a patient population with a PHQ9 score of >10 or BMI of >30, respectively.

Patients self-reported their use of a mobilization device, SOB/CP, and medication adherence. Notably, not all patients have all outcome measures recorded. Due to the inherent nature of patient-reported data, should a patient not report a measure, our data collection system was unable to capture it, likely reflecting the lower data availability rates in the PHQ-9 score and DASI. We are unable to determine the exact reason a patient may not have reported these data. To account for this, we reported the percentage of patients with data present alongside the outcomes data. The virtual PR intervention was delivered using the Carda Health platform (Carda Health, Inc), which delivered an individualized, supervised exercise program (by certified clinical exercise physiologists) with live patient vital monitoring based on center-based AACVPR standards alongside tailored education on nutrition, stress management, and medication adherence.

We investigated this cohort to understand demographics stratified by different patient-reported outcome measures including sex, race, transportation access, fixed income status, presence of hypertension, and smoking status. All of these data are patient reported outcomes measured.

We measured safety data (2023‐2024) including the number of total safety events, safety event rate (for patients who completed the program), safety events prior to PR session interventions, safety events post PR session intervention, medical team contact events/rate, emergency medical services (EMS) activation events/rate, and death (defined as a patient’s passing while in PR). Safety events were measured internally upon the occurrence of any qualifying safety event.

A safety event included but was not limited to patient complaints, serious injury, or death. Serious injury included but was not limited to hypotension, tachyarrhythmia, or CP, new mental health crisis, or suspected vulnerable adult maltreatment.

### Statistical Analysis

We calculated the difference in these outcome measures both before and after the PR intervention. Statistical testing included a 1-tailed *t* test and Cohen *d*. Cohen *d* was used to quantify the clinically meaningful magnitude of change across diverse outcomes independent of sample size, and one-tailed testing was applied given the prespecified clinical expectation that PR would result in improvement rather than harm. Our effect sizes were classified as follows: 0‐0.2=very small, 0.2‐0.5=small, 0.5‐0.8=moderate, and >0.8=large. We reported the absolute value of Cohen *d* to highlight the effect size given that mean changes were reported with directionality if relevant (+/-). For the single-tailed T score, a *P* value less than .05 was considered the threshold for significance. All analyses were performed using Microsoft Excel.

### Ethical Considerations

Upon enrollment into the PR program, patients consented to the use of their information for research purposes. All data were deidentified prior to statistical analysis. This retrospective observational study involved secondary analysis of existing, deidentified, administrative data. Because the study met the criteria for research involving minimal risk and posed no additional risk to individuals than usual care, the project was determined to be exempt from Institutional Review Board review under applicable federal regulations.

## Results

Overall, 93.0% (n=2444) of all patients who started PR completed it in accordance with the AACVPR 10-session threshold for completion.

### Primary Outcome Measures

The mean difference in maximum METs achieved was 1.44 (*P*<.001; Cohen *d*=2.51). The mean difference in DASI was 6.29 (*P*<.001; Cohen *d*=0.57). The mean difference in the PHQ-9 depression score was −1.87 (*P*<.001; Cohen *d*=0.56). The mean difference in the Sit-to-Stand test score was 2.02 (*P*<.001; Cohen *d*=0.10). The mean difference in mobilization assistive device use was −0.05 (*P*<.05; Cohen *d*=0.09). There was no significant difference in the percentage of sessions with SOB or CP (0.43%; *P*=.17; Cohen *d*=0.02).

The mean difference in resting SBP and DBP were −2.25 mm Hg (*P*<.001; Cohen *d*=0.16) and −1.64 mm Hg (*P*<.001; Cohen *d*=0.20), respectively. Medication adherence increased by 2.90% (*P*<.001; Cohen *d*=0.20). The mean difference in BMI was −0.28 kg/m² (*P*<.05; Cohen *d*=0.03). No statistically meaningful differences were observed in resting heart rate (0.11 beats per minute; *P*=.25; Cohen *d*=0.01) or differences in resting oxygen saturation (Cohen d0; *P*=.48; Cohen *d*=0.00). The mean time from enrollment to the first session was 22.6 days (Table 1).

Among patients with an initial PHQ-9 score of ≥10 (moderate depression), their preintervention mean score was 12.8 and their postintervention mean score was 7.83 (*P*<.001; Cohen *d*=1.26). Among patients with a BMI of ≥30 (BMI≥30 reflects obesity), their preintervention mean BMI was 36.4 and their postintervention mean BMI was 35.9 (*P*<.001; Cohen *d*=0.08).

**Table 1.** Pulmonary rehabilitation outcome measures.

Name	Mean difference	*P* value (1-tailed)	|Cohen *d*|	95% CI	Percentage of patient data, %
Difference in maximum METs^a^ achieved	1.44	<.05	2.506	2.427 to 2.585	100
Difference in DASI^b^	6.29	<.05	0.575	0.471 to 0.679	61
Difference in PHQ-9^c^ scoring	−1.87	<.05	0.560	0.457 to 0.663	62
Difference in average resting diastolic BP^d^	−1.64	<.05	0.202	0.123 to 0.281	100
Difference in medication adherence	2.90%	<.05	0.203	0.124 to 0.282	100
Difference in average resting systolic BP	−2.25	<.05	0.162	0.083 to 0.241	100
Difference in Sit-to-Stand test scoring	2.02	<.05	0.096	−0.010 to 0.202	56
Difference in mobilization assistive device need	−0.05	<.05	0.086	0.005 to 0.177	76
Difference in BMI	−0.28	<.05	0.025	−0.054 to 0.104	100
Difference in the percentage of sessions with SOB^e^ or CP^f^	0.43%	.17	0.018	−0.061 to 0.097	100
Difference in average resting heart rate	0.11	.25	0.007	−0.086 to 0.072	100
Difference in resting oxygen saturation	0	.48	0.001	−0.080 to 0.078	100
Average days from enrollment to the first session	22.63	—^g^	—	—	100

a MET: maximum metabolic equivalent.

b DASI: Duke Activity Status Index.

c PHQ-9: Patient Health Questionnaire-9.

d BP: blood pressure.

e SOB: shortness of breath.

f CP: chest pain.

g Not applicable.

### Secondary Outcome Measures

Regarding smoking status, 287 (11.7%) patients smoked prior to the study. Forty-four (15.3%) patients were no longer smoking after they completed virtual PR. Regarding hypertension, 1560 patients had hypertension (resting SBP>130 or DBP>90 mm Hg) prior to starting virtual PR. Of them, 600 (38.5%) no longer had hypertension after they completed virtual PR.

Regarding safety data, there were a total of 567 safety events, 380 of which occurred between 2023 and 2024 among patients who completed PR. This corresponded to a safety event rate of 0.52% (between 2023 and 2024) out of approximately 73,000 visits. Overall, 80.7% of all events were identified before the PR intervention was delivered, while 19.40% of them occurred during or after the intervention. Across all safety events, the patient’s primary provider was contacted 83.6% of the time and EMS were activated 25.2% of the time. No deaths occurred (Table 2).

**Table 2.** Safety event data.

	Outcome
Number of PR^a^ sessions (patient-matched), n	72,860
Total safety events, n	567
Total safety events (patient-matched), n	380
Safety event rate (patient-matched), %	0.52
Safety events identified prior to the intervention, %	80.70
Safety events identified during or post the intervention, %	19.40
Medical team contact events, n	471
Medical team contact rate, %	83.60
EMS^b^ activation events, n	143
EMS activation event rate, %	25.20
Deaths, n	0

a PR: pulmonary rehabilitation.

b EMS: emergency medical services.

The average patient age when starting virtual PR was 73.1 (range 30-98, mode 71) year. Furthermore, 35.8% (387/1082) of patients were male, 64.0% (693/1082) were female, and 0.3% (2/1082) reported “other” or “prefer not to answer.” Moreover, 9.3% (102/1100) of patients were African American, 82.5% (908/1100) were White, 0.9% (10/1100) were Hispanic or Latino, 1.2% (13/1100) were American Indian or Alaskan, 0.9% (10/1100) were Asian, 2.3% (25/1100) belonged to “other” racial/ethnic groups, and 2.9% (32/1100) responded “prefer not to answer.”

Regarding transportation access, 2.8% (30/1086) of patients used public transportation, 82.2% (893/1086) have a vehicle and legal license, 13.1% (142/1086) have their family or friends drive them, 1.2% (13/1086) do not have any access to any form of transport, and 0.7% (8/1086) responded “prefer not to answer.”

Regarding income status, 91.0% (960/1054) of patients reported that they are on a fixed income and 8.7% (92/1054) were not, while 0.3% (2/1054) responded “prefer not to answer.” These demographic data are reported in Table 3.

**Table 3.** Demographic data of patient population.

Characteristics	Patients, n (%)
Sex (n=1082)	
Male	387 (35.8)
Female	693 (64.0)
Other/prefer not to answer	2 (0.3)
Race (n=1100)	
African American	102 (9.3)
White	908 (82.5)
Hispanic or Latino	10 (0.9)
American Indian or Alaskan	13 (1.2)
Asian	10 (0.9)
Other	25 (2.3)
Prefer not to answer	32 (2.9)
Transportation access (n=1086)	
Public transportation	30 (2.8)
Own vehicle with legal license	893 (82.2)
Family/friends drive them	142 (13.1)
No access to transportation	13 (1.2)
Prefer not to answer	8 (0.7)
Income status (n=1054)	
Fixed income	960 (91.0)
Not fixed income	92 (8.7)
Prefer not to answer	2 (0.3)

## Discussion

Given the significant barriers in patient access to brick-and-mortar PR, primarily driven by transportation challenges and geographic distance, virtual PR may be a promising solution to overcome these barriers [5]. This study demonstrates the efficacy of virtual PR delivered by a digital platform on medical, behavioral, and functional outcome measures. Especially considering the underappreciated influence of mental health and physical inactivity on COPD, the association between virtual PR and these factors is a promising finding [13].

Notably our completion rates of PR are nearly identical to a 2021 Cochrane review that identified a 93% completion rate across 1904 participants for virtual PR [9].

By evaluating the Cohen *d* score, the following outcome measures had the strongest effect sizes: maximum METs achieved, improvement in the PHQ-9 score, improvement in the DASI, and improvement in the DBP. Further, the marked decreases in smoking rates and hypertension prevalence were additional promising findings. Further, the high prevalence of patients on a fixed income suggests that virtual PR may be a feasible mechanism to deliver virtual PR to them.

It is also encouraging that not only was there a low safety event rate, but also over 80% of these events were identified before any intervention was delivered on any given day, possibly avoiding or identifying a potentially dangerous event.

Mosher et al [11] identified that pulmonary rehabilitation after COPD exacerbations is a powerful tool to reduce health care costs and improve quality-adjusted life expectancy. As Medicare and commercial payers consider reimbursement eligibility and strategies for digital therapies such as virtual PR, support for the efficacy of virtual PR will inform policymakers and the industry in their determinations in terms of access to this therapy and reimbursement. It will also be similarly important for physicians to acknowledge the opportunity, feasibility, effectiveness, and safety of virtual PR among the patients they care for; therefore, continuing to investigate this mode of care delivery will be vital. Therefore, continuing to establish standardized quality outcomes on virtual PR will be needed to scale it safely for patients across the country.

The small yet significant effect size of PR associated with a reduction in SBP/DBP may lead to a reduction in medical costs through a decreased risk of developing cardiovascular disease and stroke. However, capturing this cost reduction may be a challenge given the relatively longer timeline for preventing disease and the relatively short timeline over which a single patient is on a single insurance plan. PR may also lead to increased revenue for Medicare Part C plans by addressing the “controlling blood pressure” HEDIS (Healthcare Effectiveness Data and Information Set) measure.

PR also had a small yet significant effect size associated with an improvement in medication adherence. This may also lead to a reduction in medical costs by preventing the complications associated with medication nonadherence. Further, PR may also lead to increased revenue by addressing the Part D quality measures of “medication adherence” for diabetes, hypertension, and hypercholesterolemia.

PR had a medium, significant effect size associated with reductions in PHQ-9 scores of all patients and a particularly very large effect size associated with reductions in PHQ-9 scores for those with PHQ-9 scores of >10. This reduction in PHQ-9 scores suggests that virtual PR is associated with improvements in depression. This may lead to additional savings as a recent cohort study with patients insured by employee-sponsored plans found that the return on investment was 1.9 times the cost [13]. With increasing recognition that improving behavioral health leads to better health for those with chronic disease, one may imagine that these improvements in PHQ-9 scores may drive improved chronic disease management, potentially decreasing hospitalizations and emergency department visits.

The very strong and significant effect size of PR associated with improved achievement of maximum METs is consistent with the expected physiological improvement in cardiorespiratory capacity driven by virtual PR. However, this finding may be confounded by the nature of the protocol of PR, which inherently includes increased METs over time. This, combined with the moderate effect size of virtual PR associated with improvements in the DASI, reflecting improved functional outcomes, supports the expectation that patients may be more able to live independently in the community rather than be reliant on acute or long-term care services: a potential opportunity for improving care quality and reducing cost.

This study is limited by its retrospective, nonrandomized design and the use of patient-reported outcome measures in addition to objective data. Further, selection bias, lack of a control group, and heterogeneity in the comorbidities of the patient populations also serve as limitations to the study. Future studies may consider using a disease-matched RCT design to further elucidate the impact of PR. Additionally, due to the nature of patient-reported outcomes, our results regarding smoking cessation and medication adherence are subject to recall and social desirability biases.

Virtual PR may be a useful tool in delivering PR care to a fixed-income patient population with chronic respiratory disease: this would not only improve their mental health, functional status, and health status but also deliver care with a very low safety event rate. This intervention could be useful in addressing the nationwide massive underuse of PR in the United States across both rural and urban populations that have sufficient connectivity. While these data are promising in their support of virtual PR in improving pulmonary, functional, and behavioral health, further investigation is needed to understand how it performs and improves access compared to brick-and-mortar PR. The bar for improving adherence to recommended PR is quite low (2.7% of Medicare population within 12 mo of eligible diagnosis); therefore, there is massive opportunity for impact and improving the quality of pulmonary care nationwide. There are likely many barriers to this: real and perceived cost, travel, provider/patient awareness, and competing priorities to name a few. Overcoming these barriers will be valuable levers to improving uptake and successful completion of PR at the national level.

## Abbreviations

AACVPR: American Association of Cardiovascular and Pulmonary Rehabilitation
COPD: chronic obstructive pulmonary disease
CP: chest pain
DASI: Duke Activity Status Index
DBP: diastolic blood pressure
EMS: emergency medical services
HEDIS: Healthcare Effectiveness Data and Information Set
MET: metabolic equivalent
N/A: not applicable
PHQ-9: Patient Health Questionnaire-9
PR: pulmonary rehabilitation
RCT: randomized controlled trial
SBP: systolic blood pressure
SOB: shortness of breath
